# Increasing the Detection Limit of the Parkinson Disorder through a Specific Surface Chemistry Applied onto Inner Surface of the Titration Well

**DOI:** 10.3390/jfb3020298

**Published:** 2012-04-18

**Authors:** Caroline Mille, Dominique Debarnot, Willy Zorzi, Benaïssa El Moualij, Arnaud Coudreuse, Gilbert Legeay, Isabelle Quadrio, Armand Perret-Liaudet, Fabienne Poncin-Epaillard

**Affiliations:** 1 Département Polymères, Institut des Molécules et Matériaux du Mans, Colloïdes et Interfaces, Lunam Université, UMR Université du Maine—CNRS No. 6283, Avenue Olivier Messiaen, Le Mans Cedex 72085, France; Email: caroline.mille.etu@univ-lemans.fr (C.M.); Dominique.Debarnot@univ-lemans.fr (D.D.); 2 Centre de Recherche sur les Protéines Prion, Institut de Pharmacie, B36, No. 1 avenue de l’Hôpital, Liège 4000, Belgium; Email: willy.zorzi@ulg.ac.be (W.Z.); b.elmoualij@ulg.ac.be (B.E.M.); 3 CTTM, 20 rue Thalès de Milet, Le Mans 72000, France; Email: acoudreuse@cttm-lemans.com (A.C.); gilbert.legeay@neuf.fr (G.L.); 4 Centre Mémoire de Ressources et Recherche, Service de Neurobiologie, Groupement Hospitalier Est du CHU, 56 bd Pinel, Bron cedex 69677, France; Email: isabelle.quadrio@chu-lyon.fr (I.Q.); armand.perret-liaudet@chu-lyon.fr (A.P.-L.)

**Keywords:** amphiphilic molecules, cold plasma, ELISA titration, α-synuclein protein

## Abstract

The main objective of this paper was to illustrate the enhancement of the sensitivity of ELISA titration for neurodegenerative proteins by reducing nonspecific adsorptions that could lead to false positives. This goal was obtained thanks to the association of plasma and wet chemistries applied to the inner surface of the titration well. The polypropylene surface was plasma-activated and then, dip-coated with different amphiphilic molecules. These molecules have more or less long hydrocarbon chains and may be charged. The modified surfaces were characterized in terms of hydrophilic—phobic character, surface chemical groups and topography. Finally, the coated wells were tested during the ELISA titration of the specific antibody capture of the α-synuclein protein. The highest sensitivity is obtained with polar (Θ = 35°), negatively charged and smooth inner surface.

## 1. Introduction

Parkinson’s disease is one of the most important neurodegenerative diseases after Alzheimer’s . It affects 1% of 50–65 years old person, mainly males [1]. Its evolution becomes gradually more and more marked in movements and brain until the death of the patient. The first symptoms were described in 1817 as a generalized trembling paralysis [2,3]. The α-synuclein protein, located in the central nervous system may be involved in its pathogenesis [4]. It has a regulating role in the stability of the cell membranes and in the neuronal plasticity, related to the mechanism of cellular answer of memorization and learning [5,6,7]. This protein also has a role of so-called chaperon molecule and prevents its irreversible aggregation that regulates associations and liberation of molecules of interest [8]. A too high concentration of this protein in cellular medium leads to an alteration of its conformation from a helicoidal state to a ß sheet state [9] that causes the degeneration and the apoptosis of neurons, then the ageing and the degeneration of nervous cells [10,11]. The alteration mechanism is still unknown, and probably corresponds to several patterns. Environmental factors such as the presence of heavy metals and pesticides, as well as a cranial traumatism may exacerbate the disease by about 70% [12,13]. The key points are multiple and deal with the complex procedures of protein extraction or recombinant proteins synthesis together with the lesser number of available capture or detection antibodies. The lack of sensitive detection is also another key-point. To our knowledge, no ELISA system allows diagnosing this disease in an *ante-mortem* state because in the first years of the disease, the concentration of infectious agents is too weak (femtogram per milliliter) to be titrated. In this paper, a decrease of the detection threshold by directly modifying the inner surface of the wells of Enzyme Linked ImmunoSorbant Assay (ELISA) is proposed. This surface modification based on plasma activation and amphiphilic molecules coating allows creating hydrophilic charged surfaces sensitive to the α-synuclein protein antibody attachment.

## 2. Results and Discussion

The biocompatibility of hydrophobic polymer surfaces such as PP, used in the biomedical field is rather a challenge [14,15]. The interactions between the substrate and the biomolecule are mostly controlled by chemical, electrostatic, Van der Waals, hydrogen bond interactions (hydrophilic-hydrophobic balance and charge effect), by the mechanical anchoring (surface roughness) that may enhance the detection threshold of ELISA titration through a better capture antibody attachment. Therefore, functionalization of the inner PP surface is studied thanks to different processes using environment-friendly wet chemistry of amphiphilic molecules and plasma chemistry [16,17,18,19,20]. 

**Scheme 1:**
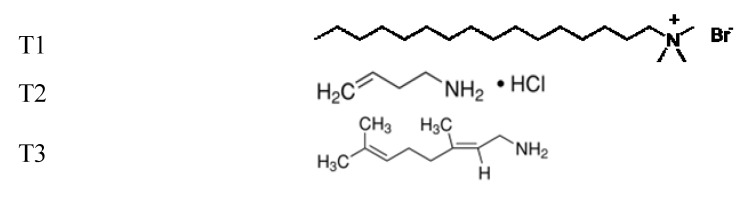
the grafting of molecules bearing amino groups.

Plasma chemistry acts as an activation step allowing the formation of reactive species onto PP surface, then the grafting of amphiphilic molecules after dipping the plasma-modified surface in such component solutions. The topography, hydrophilic and charge characters are induced and controlled by such molecules deposition. Discussion will be focused on Ar and He plasma treatments [21] and the grafting of molecules bearing amino groups [22,23,24,25] as Scheme 1.

In our previous work [19], the efficiency of the surface treatment was studied with another neurodegenerative protein (Tau protein). One of the chosen surface modifications, *i.e*., T3 coating enhances the ELISA titration. Here, the study is extended to another neurodegenerative, the α-synuclein. The plasma parameters (duration *t*; power, gas flow and chemical nature summarized as the *W/FM ratio*) controlling the densities of reactive species that bombard the surface; the coating parameters (concentration *c*, duration *t*) adjusting thickness and organization of the thin layer were optimized. Then, all the modified PP surfaces were characterized thanks to wettability measurements, XPS, AFM analyses. 

### 2.1. Cold Plasma Activation of PP Surface

The plasma technique allows the modification of material surface without altering its bulk properties [26]. Gases most often used to activate a surface are argon and helium leading to a homogeneous reactive surface [27]. These plasma phases are compared in order to obtain the most attractive surface towards the further coating step, with a non-dispersive surface energy around 40 mJ·m^−2^. 

**Figure 1:**
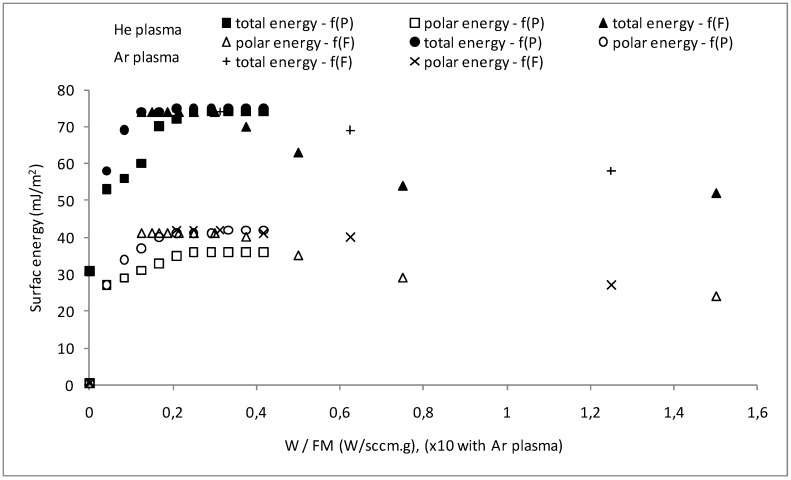
Dependence of the total and non-dispersive energies of plasma-treated PP on *W/FM* ratio [(f(P): *F* = 60 sccm, *t* = 2 min, *p = 10*^−2^ *mbar*; f(*F*): *P* (He) = 60 W and *P* (Ar) = 50 W, *t* = 2 min, *p =* 10^−2^ mbar)]

The evolution of the hydrophilic character of such a modified surface, described as the variation of water contact angle, according to the values of *W/FM* Yasuda ratio is given in Figure 1.

It appears that the overlapping of the power and flow parameters sets, leading to the same values of *W/FM*, almost induces the same values of surface energies. Increasing the Yasuda ratio leads to a variation of surface energies passing through a maximal value for both Ar and He plasma treatments. This plateau takes place for *W/FM* values comprised between 0.208 and 0.375; 0.012 and 0.042 W·sccm^−1^·g^−1^ respectively for He and Ar plasmas. The corresponding power and flow ranges are 60–100 W and 50–120 sccm for He plasma; 30–100 W and 30–60 sccm in case of Ar plasma. These values of plasma energy allow an increase of the PP surface energies. For example, the non-dispersive (polar) component, negligible for the virgin sample, reaches the value of 40 mJ·m^−2^. Then, the observed decrease of surface energies for higher Yasuda ratio should be associated to the competitive reaction, *i.e.*, degradation of the polymeric material, already noticed for plasma treatment of polymers [28]. 

**Figure 2:**
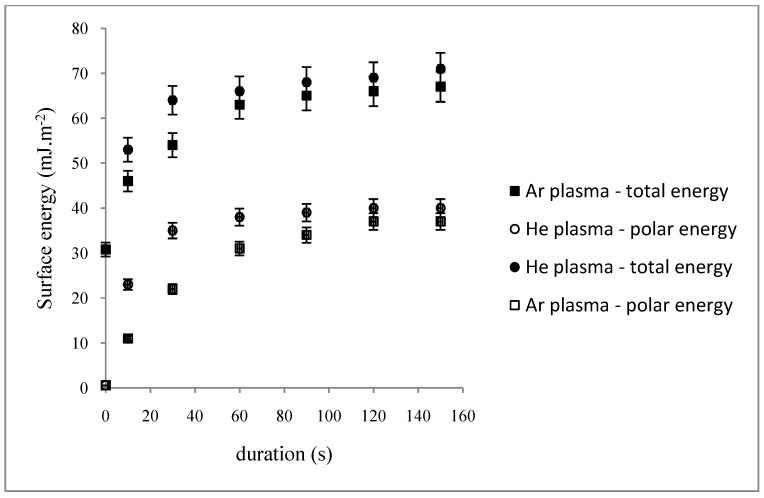
Dependence of the total and non-dispersive energies of plasma-treated PP on duration (*p =* 10^−2^ mbar, *W/FM (He)* = 0.15, *W/FM (Ar)* = 0.025.

The dependence on duration for specific values of *W/FM* was also studied (Figure 2). The surface energies towards the plasma-treatment duration first slowly increase, then reach a plateau at 60 s (*Θ_H_2_O_* ≈ 27°, *γ^t^* ≈ 66 mJ·m^−2^, *γ^nd^*≈ 38 mJ·m^−2^) and at 90 s (*Θ_H_2_O_* ≈ 34°, *γ^t^* ≈ 63 mJ·m^−2^, *γ^nd^* ≈ 31 mJ·m^−2^) respectively for He and Ar plasma phases (Figure 2). In such conditions, both plasma phases lead to a moderate hydrophilic PP surface. Further experiments will be run under these chosen plasma parameters. 

One of these experiments was focused on the repeatability of the plasma-treatment of PP surfaces since these treatments will be scaled up. A good reproducibility requires choosing the argon plasma as the activation step. Indeed, the obtained polar surface energies of 8 samples present a variation of 28.5 mJ·m^−2^ with a corresponding domain of water contact angle from *Θ* = 46.2° to *Θ* = 27.7°. The treatment of PP surfaces with argon plasma is not reproducible. On the other hand, the helium plasma treatment leads to better stability and repeatability of the measures. Indeed, the average values of polar surface energy is around 39.2 mJ·m^−2^ (*Θ* = 24.4°) with an average error of 1.2 mJ·m^−2^. The helium plasma is shown to be more efficient. Therefore, all PP samples were activated in He plasma in such conditions: *W/FM* = 0.15 and *t* = 60 s then dipped in the different amphiphilic solutions (T1, T2, T3).

### 2.2. Amphiphilic Coatings

**Figure 3:**
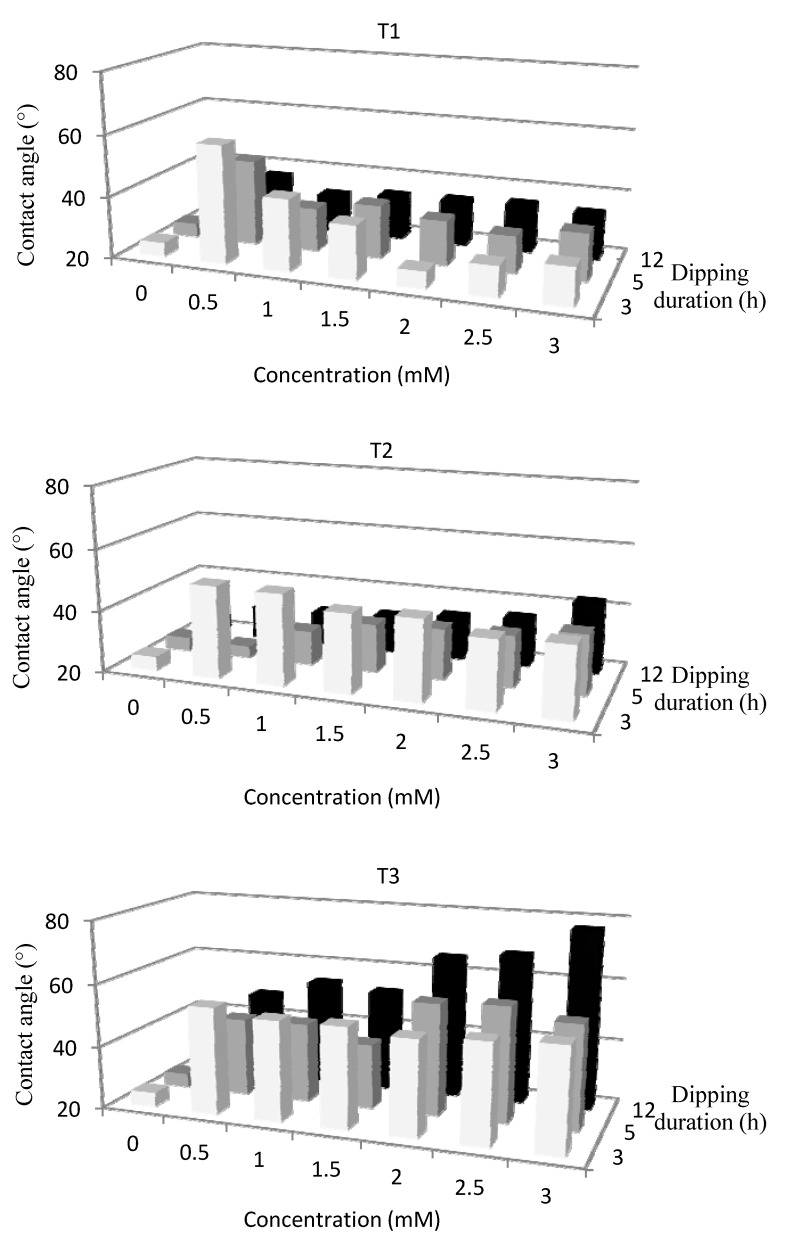
Dependence of the wettability of coated and plasma-treated PP surface on concentration of amphiphilic molecules and dipping duration (*p =* 10^−2^ mbar, *W/FM (He)* = 0.15, *t (He)* = 60 s).

Since the specific auto-organization behavior of such molecules, the coated molecule should have a certain orientation with the hydrophilic head attracted by the hydrophilic plasma-treated surface. A multi-layers deposition is expected with sequences head-tail-tail-head association leading to a hydrophilic extreme surface of the coated substrate. Besides the thermodynamic requirement on interfacial energy between the substrate and the amphiphilic molecules, and between two molecules, the homogeneity of the coating onto substrate depends mostly on two factors corresponding to the molecules concentration in aqueous solution and the dipping duration. In a first step, these dependences have been studied at room temperature and followed by contact angle measurement (Figure 3). 

Other surface characterizations such as XPS, AFM giving details on chemical composition and roughness will described in the following section for few specific surfaces. 

At 25 °C with 3 or 5 h of dipping, the H_2_O contact angle onto T1-coated surface, after increasing at very low T1 concentration, is decreasing while the T1 solution becomes more concentrated. With 3 mM concentration, the contact angle reaches the values of 48° and 35° respectively for 3 and 5 h of dipping. The initial increase should correspond to an incomplete layer or a well organized monolayer leading to hydrophobic tail at the extreme surface. With such dipping conditions, hydrophilic surface is obtained with T1 concentration higher than the critical aggregation concentration (CAC) equal to 0.83 mM [29] and determined in our experiment as 1.05 mM. In addition, beyond the CAC, the evolution of the contact angle values, not shown here, corresponding to an immersion of plasma-treated PP during 3 h, shows an important fluctuation probably indicating that the coated layer is still disoriented without any specific organization. On the contrary, above the CAC, 5 h of immersion allows the surfactant molecules to stabilize themselves in the medium and to be in equilibrium state between the molecules remaining in solution and those adhering onto the plasma-treated PP surface. The 12 h immersion curve presents for any concentration, a contact angle value almost always identical (around 34°). This duration seems to induce a nice and smooth interface between the modified substrate and surfactant solution. Thus, 12 h as duration and 1mM as T1 concentration will be used for the treatment of ELISA well inner surface.

Now considering T2, an immersion of 3 h does not seem to be appropriate. Although the increase of the molar concentration involves a decrease of the contact angle; it remains however too high for our purpose. With 5 h or 12 h of immersion, a same behavior is noticed when the molecular concentration increases. The water contact angles vary from 24° and 31°, respectively for 5 h and 12 h, at 0.5 mM concentration; to 39° and 43° with a 3 mM concentration. Thus, 5 h as duration and 1 mM as T2 concentration will be used for the treatment of inner surface of ELISA well.

The last experiments undertaken with T3 induce contact angle values quite higher than those obtained with the treatments T1 and T2. Coating obtained with 3 h of immersion shows an almost constant contact angle close to 46° whatever the T3 molar concentration is. On the other hand, the graphs associated to longer immersion (5 h and 12 h) present similar behaviors according to the molar concentration. The higher this concentration is, the higher the contact angle is; 53° and 77° with a concentration of 3 mM for 5 h and 12 h immersion, respectively. This can be due to a reorganization of the amphiphilic molecules in the medium in order to minimize energies. Indeed, the small quantity of free molecules remaining in aqueous medium involves a decrease of the surface tension of the solution. Thus, to counterbalance this effect, the amphiphilic molecules present either their hydrophilic part or their hydrophobic chain. Compared to the plasma-treated surface, the PP surface becomes more hydrophobic and the value of the associated contact angle increases. Thus, 5 h as duration and 1.5 mM as T3 concentration will be used for the treatment of inner surface of ELISA well.

The ageing of such modified surface must be controlled since, in hospital surrounding the immunoenzymatic analyzes are not systematically carried out the same day as blood aliquot sampling. Accordingly, a series of eight samples for each treatment T1, T2 and T3 was carried out followed by measurement of the contact angle each week during 2 months. The contact angles of PP-T1 and PP-T2 vary little over that duration. T1 treatment gives rise to an average value of the contact angle of 34° with an error of 1.8° and T2 treatment, a contact angle of 36° with average error of 0.8°. Therefore, they have both a very good stability over time. In contrast, the ageing of treatment T3 has a shift over time, particularly after 5 weeks. The deposition of thin films seems to deteriorate over time. This was confirmed by the averaged values of contact angles that the error is significant: 49° ± 3.6°. In addition, ageing of coatings were also evaluated in enzyme immunoassay detection and confirm the results obtained here.

### 2.3. Physicochemical Characterization of Coatings

In each case, samples whose surface has been only activated are denoted PP-He, those dipped in T1, T2 or T3 solutions denoted as PP-T1, PP-T2, PP-T3 are compared to the untreated PP support. Table 1 summarizes the wettability and surface energy of the different modified surfaces according to the experimental conditions [30]. 

**Table 1:** Wettability, surface energy, elemental composition and streaming potential of the different modified surfaces.

	Θ _H__2O_ (°)	γ^t^ (mJ·m^−2^)	γ^nd^ (mJ·m^−2^)	γ^d^ (mJ·m^−2^)	C1s	N1s	O1s	ζ (mV, pH 7.4)
PP	98.6	30.8	0.6	30.2	99.1	-	0.9	−36.4
PP-He	27.7	66.2	36.8	29.4	76.4	1.8	21.8	-
PP-T1	34.0	61.0	40.4	20.4	93.3	1.4	5.0	−53.9
PP-T2	36.4	60.3	34.7	25.6	88.5	2.2	9.3	−61.2
PP-T3	45.4	55.1	27.5	27.6	89.2	2.0	8.8	−43.6

The hydrophilicity of the modified surfaces increases after treatment. The presence on the PP surface of functional thin films can, therefore, reduce its hydrophobicity. Though the non dispersive energy of coated samples is in the same order or lower than that of plasma-treated sample, it has been demonstrated in a previous work, that the sensitivity of ELISA titration is much more enhanced with a coating [24] indicating that the hydrophilicity is not the only pertinent parameter that influences the antibody attachment.

The surface chemistry of untreated and treated materials was evaluated by XPS (Table 1). The selected PP being 99% pure is mainly composed of carbon atoms and hydrogen atoms. The oxygen traces can be either detected in the polymer, or may arise during handling manipulation of the sample. Furthermore, the He plasma-treated PP surface shows some nitrogen and oxygen atoms in proportions of 1.8% and 21.4% respectively corresponding to hydroxyl, amine, carbonyl and carboxylic groups. The helium plasma can thus activate the PP surface and create highly reactive free radicals which can then interact with air or latter with the amphiphilic molecules. After the deposition of thin films, the atomic carbon percentage for each treatment is greater than that obtained for the plasma-activated surface. The percentages increase from 76.4% (PP) to 93.3% (PP-T1), 88.5% (PP-T2) and 88.95% (PP-T3) respectively. Even if the N/C ratio of coated PP surfaces found by XPS analysis does not correspond to the theoretical one (0.056, 0.25 and 0.111 respectively for PP-T1, PP-T2 and PP-T3), the larger increase of N% noticed for the shorter hydrocarbon chain (T2) gives evidence of coating. The coating affects also the presence of oxygen at the surface but does not completely erase it. Nevertheless, the percentage values are not zero but 5.0%, 9.3% and 8.8% for PP-T1, PP-T2 and PP-T3, respectively. That may due to the presence of quenched water or due to a deeper analyze depth penetration than the layer thickness. Moreover, the presence of Br in PP-T1 also confirms the deposition even if, once again, the Br/C ratio differs from the theoretical value (Br/C = 0.015). This fact could be explained by a deeper analyze depth. In addition, during the immersion and drying, the ion may be removed. The treated surfaces were also analyzed in different locations to verify the homogeneity of the treatments. The results obtained by XPS show that treatments are homogeneous. Indeed, for example, PP-T2 elemental composition is almost identical; the atomic carbon percentages vary from 88.5% to 87.9%.

To assess the surface topography of samples, SEM and AFM microscopies have been completed. EDX spectra were also performed. Each EDX spectrum, not shown here, has a major peak around 0.25 KeV corresponding to the carbon atom whose intensity is decreasing after the plasma treatment and the coating processes. A second peak also appears at 0.5 KeV corresponding to the oxygen atom with functionalized sample, as already seen by XPS. The EDX spectrum of PP-T1 also presents a peak around 1.55 KeV assigned to the bromine atom and confirming the presence of the surfactant molecule on the surface of PP. Nitrogen atom was not detected probably due to a lack of sensitivity. Compared to the virgin surface, the SEM image (not given here) obtained after metallization of the PP-He surface shows a smooth and homogeneous surface while the images corresponding to the T1, T2 and T3 coated substrates confirm that a homogeneous deposit is formed on the surface of plasma-treated in presence of some of aggregates. 

The AFM pictures (not given here) evaluate homogeneity of the surfaces but also the roughness in the µm scale. The virgin PP image presents some scratches. The plasma-treated one’s appears smoother while the coated PP one’s are more uniform even if some aggregates are spread relatively uniformly across the analyzed surface. Their average size is around 15 nm. Moreover, the presence of these aggregates can increase the surface roughness. PP-T1 has a roughness of 3.2 nm, *i.e.*, twice the initial roughness of the PP and PP-T2 has a roughness of 4.5 nm or three times that of PP control. However, the roughness is still almost negligible for polymeric materials. 

Table 1 shows the *ζ* potential of PP and functionalized PP-T1, PP-T2, PP-T3 at pH 7.4, working pH of ELISA titration of α-synuclein. All modified and virgin surfaces present an isoelectric point lower than the pH (7.4) of the buffer solution for the ELISA titration [31]. Therefore, the biofunctionalized wells surfaces will be negatively charged during all the protocol of ELISA titration. Since PP-T3 is a neutral molecule containing amino groups that may be ionized in contact of polyelectrolyte solution, its surface presents, in function of the pH, the lowest value of *ζ* potential of the modified surfaces but still higher than virgin PP one’s. The comparison of the results of titration sensitivity (see below) and surface properties of modified wells (Table 1) shows that the titration of α-synuclein with the lowest isoelectric point requires inner surfaces of the well with high surface energy, the lowest *ζ* potential and isoelectric point. In a same manner, its dosage sensitivity is always the lowest with virgin well possessing hydrophobic properties, low *ζ* potential and highest isoelectric point. Therefore, one may conclude that high sensitivity is reached with surface chemistry leading to a high negatively value of *ζ* potential at pH 7.4. On this charge criterion, PP-T2 must be selected.

If the surface charge of the well will control the interaction with the protein, the ionic strength effect should also considered, especially in the first step of ELISA titration corresponding to the capture antibody coating. The coating of antibodies of the α-synuclein are run in PBS at pH = 7.4 with an ionic strength around 0.170 mol·L^−1^. 

### 2.4. Illustration of the Modified PP as New ELISA Wells Used for the Titration of α-Synuclein Protein

**Figure 4:**
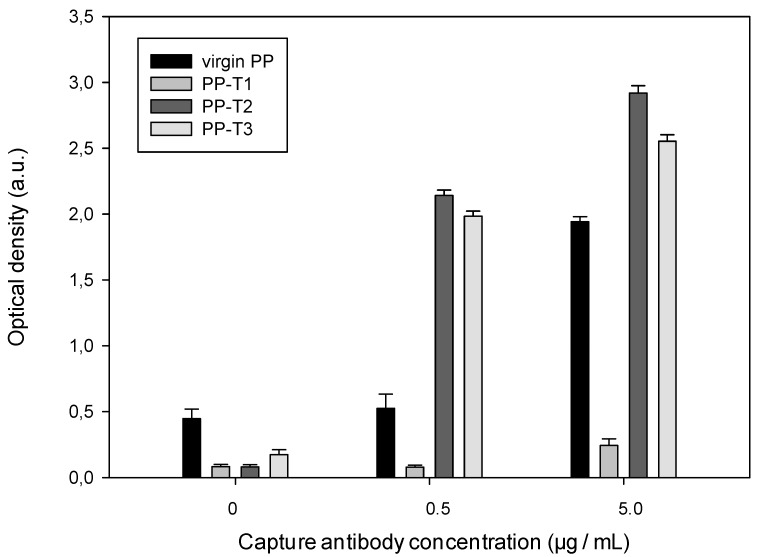
Dependence of the antibody affinity for capturing the α-synuclein on the well inner surface nature ([detection antibody] = 0.3 mg·mL^−1^).

**Figure 5:**
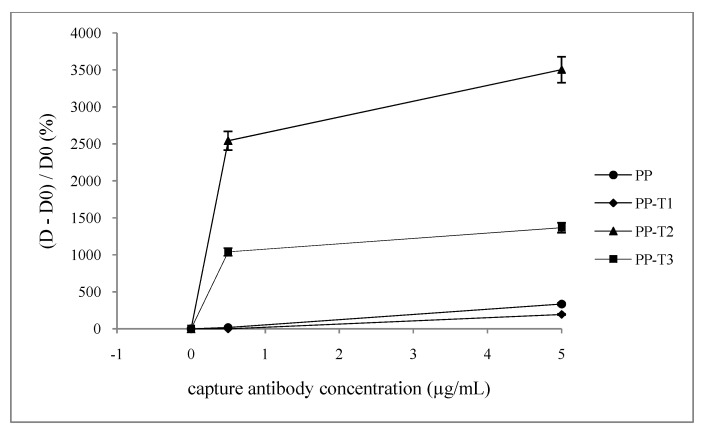
Dependence of the *(D* −*D0)/D0* ration on the well inner surface nature and the capture antibody concentration ([detection antibody] = 0.3 mg·mL^−1^).

This ELISA titration gives evidence of the presence of specific antibody or antigen. The main aim here is to improve the binding of the antibody in order to increase the amount of detected antigen, ie to improve the detection sensitivity, so only the attachment of the capture antibody on the treated substrates was evaluated. Figure 4 and Figure 5 show the evolution of either the optical density (*D*) obtained withT1, T2 and T3 surfaces or the ratio *(D* − *D0)/D0* where *D0* represents the obtained optical density without capture antibody for the different surfaces (virgin PP, T1, T2 or T3). 

It can be noticed differences in affinity of the primary antibody for the different considered surfaces. The detection and capture antibodies have the same affinity and differ only by the attachment of horseradish peroxidase onto the secondary antibody. Thus, when the capture antibody concentration is zero, the affinity of detection antibody towards the modified surface is straight evaluated. The optical density values (Figure 4), in the absence of primary antibody, show that the treated substrates in presence of blocking agent do not fix the secondary antibody. Indeed, the optical density of the treated substrates is lower than one’s of virgin PP substrate. In presence of antibody, while the PP-T1 doesn’t enhance but reduces the detection, the T2 and T3 substrates lead to higher optical densities and higher quenching of the detection antibody than with virgin PP.

When determining the *(D-D0)/D0* ratio, so-called sensitivity (Figure 5), it appears that the PP-T2 is the most sensitive. In contrast, the PP-T1 has no affinity for the capture antibody since the values of optical densities obtained for each concentration does not exceed the background noise of virgin PP control. In the latter case, the sensitivity threshold is around 40%/(µg·mL^−1^) little lower than with virgin PP (68%/(µg·mL^−1^)). This may be due to the presence of ammonium group having a positive charge that could be causing repulsion towards the capture antibody. Both PP-T2 and PP-T3 enhance the sensitivity of the titration 510%/(µg·mL^−1^) and 190%/(µg·mL^−1^) respectively and even, at low antibody concentration (0.5 µg·mL^−1^) the saturation seems to be effective. It should be noticed in Figure 5 that graphs profiles are sharp at low concentrations. Other intermediate concentrations of antibody should be tested; however the available antibody quantities do not allow us to run these titration experiments. From these two graphs, one may conclude that an *ante mortem* titration of α-synuclein may be available with such modified inner surface well. It appears that high sensitivity is reached with surface chemistry leading to a high negatively value of *ζ* potential at pH 7.4. Some direct ELISA titrations of α-synuclein were also run and confirmed the previous conclusion: highest sensitivity is also obtained with PP-T2.

## 3. Experimental Section

### 3.1. Material*s*

The polypropylene supports (7 cm^2^ plates and strips of eight wells, Θ_H2O_ = 99°, γ^t^ = 30.8 mJ·m^−2^, γ^nd^ = 0.6 mJ·m^−2^) were manufactured by the company EUDICA (Annecy, France). PP samples were washed in ethanol under ultrasound for 15 min and dried 5 hours under a laminar flow hood.

The hexatrimethylammonium bromide (T1, M = 364.46 g/mol), 3-buten-1-amine hydrochloride (T2, M = 107.58 g/mol) and trans-3,7-dimethyl-2,6-octadien-1-allynamine (T3, M = 153.26 g/mol) are commercial products (Aldrich) used without further purification. 

### 3.2. Cold Plasma Treatment

The radiofrequency (RF) plasma reactor was described in [31]. The experiment was performed by optimizing the parameters influencing the treatment: gas nature, discharge power (*P*), gas flow (*F*), duration of treatment (*t*). The ratio (*W/FM*) as defined by Yasuda [32] summarizes the both variation of flow or power parameters. 

The substrate was introduced into the reactor chamber. The pressure of the chamber was initially 10^−6^ mbar. Helium or argon (purity > 99%, Alcatel) was introduced into the chamber, then the other parameters were fixed. After certain duration, the plasma was turned off and the vacuum was broken down to atmospheric pressure.

### 3.3. Amphiphilic Coating

The products were dissolved in distilled water under ultrasonic for 20 min at 37 °C, except for T3 where 10% V/V ethanol were added. Following exposure to plasma, PP was immersed in aqueous solution of T1, T2 and T3 (1 mM).

After certain duration, the sample was removed and dried for 5 hours under a laminar flow hood. It was then packaged under ambient atmosphere in a sterile polyethylene bag.

### 3.4. Wettability Measurement

In order to evaluate the wettability of surfaces, contact angle measurements were carried out with several liquids on a goniometer from RAME-HART.inc (model: 100-00-230). Then, their non dispersive (γ^nd^), total (γ^t^) energies of each surface were calculated using the Fowkes-Dupre-Young method on the average values of contact angles [33]. Water as polar liquid and diiodomethane as a non polar liquid were used. Their values of energies are as followed:

Water: γ^t^ = 72.8 mJ/m^2^ γ^nd^ = 21.8 mJ/m^2^ γ^d^ = 51.0 mJ/m^2^

Diiodomethane: γ^t^ = 50.8 mJ/m^2^ γ^nd^ = 49.5 mJ/m^2^ γ^d^ = 1.3 mJ/m^2^

The value of the contact angle was the average of six measurements.

### 3.5. XPS

Analyses were performed at the Josef Stefan Institute in Ljubljana (Slovenia) on TFA XPS spectrometer equipped with Al anode (Kα = 1486.6 eV) at 45° to the surface. All spectra were referenced from the main C1s peak at 284.9 eV. 

### 3.6. AFM

Measurements were performed in Ljubljana on a device type Solver PRO, NT-MDT in tapping mode. The samples were scanned in air over an area of 5 × 5 µm^2^ with a silicon tip with a force constant of 10 Nm^−1^ and a resonance frequency of 170 kHz. The average roughness was obtained from 5 different areas.

### 3.7. Streaming Potential

The measurements were performed on the apparatus ZêtaCAD (CAD Instrument Company). The temperature experiment was consistently maintained at 25 °C. Two samples previously treated under the same conditions were placed vis-à-vis the analysis cell (25 × 50 × 5 mm) at a distance of 1 mm. A pH 7.4 buffer solution was prepared from ultrapure water and 0.9% sodium chloride, the pH being adjusted with sodium hydroxide. The pH was measured with a pH meter HI–8014 (HANNA Instruments). The solutions were introduced in the analysis cell at a constant pressure of 20 mbar. The samples were rinsed with ultrapure water. Each experiment was replicated three times. To ensure the absence of hysteresis, each pH range was performed in ascending and descending. The calculation of *ζ* potential was run using the software ZêtaCAD (resolution 800 × 600 screen drivers: SVGA) and which connected the meter. The value of *ζ* potential restraint was the average of six values.

### 3.8. ELISA Titration

Experiments run in Liege or in Lyon were performed on a spectrophotometer Bio-Tek Instruments, EL-312th microplate Bio-Kinetics reader. Plastic well surfaces were precoated with 0.3 mg/mL of detection antibody-HRP diluted in carbonate buffer (pH 7.2) at 4 °C overnight. The next day, the wells were emptied, washed 3 times (washing buffer: 0.5 mL/L Tween 20). Peroxidase-conjugated streptavidin (Dako; diluted 1:7500) was added to each well and incubated for 30 min at room temperature. After 5 washes in PBS, the residual peroxidase activity was measured by means of reaction with a solution containing equal amounts, by volume, of 3,3’,5,5’-tetramethylbenzidine and H_2_O_2_ (BD PharMingen). After incubation for 30 min in the dark at room temperature, the reaction was stopped by addition of 1 mol/L H_2_SO_4_. The absorbance of the reaction mixture was measured at 450 nm with an automatic reader instrument.

## 4. Conclusions

Thanks to the plasma activation and the amphiphilic molecules coating, the PP well inner surface becomes more hydrophilic and/or charged without altering the roughness of the polymeric material. However, some aggregates whose size corresponds to a few ten nm appear depending on the chemical nature of the amphiphilic molecule. The plasma activation depends on the Yasuda ratio and the He plasma-treated surface are selected for the further coating step. Three different amino layers composed of one hydrophobic chain and hydrophilic head were deposited onto the plasma-treated surface. One of these is charged. Except the ionized one, the T1 layer, the coatings were shown to be homogeneous and stable on storage duration. ELISA titration of the capture antibody of α-synuclein run in the modified well indicates that strong and high affinity is obtained for a surface with a short hydrophobic chain and hydrophilic head that can be ionized during one of the steps of the titration protocole, such as PP-T2 or T3.

Since the described surface modification of titration wells is dependent on the physicochemical properties (hydrophilic character, charge effect…) of the biomolecules, either capture, detection antibodies or antigens, this procedure could achieve an enhancement of the immunoassays with auto-antibodies multimers.
